# Ponatinib modulates the metabolic profile of obese mice by inhibiting adipose tissue macrophage inflammation

**DOI:** 10.3389/fphar.2022.1040999

**Published:** 2022-11-15

**Authors:** Zhuomiao Lin, Xiaochun Lin, Ying Lai, Congcong Han, Xinran Fan, Jie Tang, Shiqi Mo, Jiahui Su, Sijia Liang, Jinyan Shang, Xiaofei Lv, Siwan Guo, Ruiping Pang, Jiaguo Zhou, Tingting Zhang, Feiran Zhang

**Affiliations:** ^1^ Department of Pharmacology, Cardiac and Cerebral Vascular Research Center, Zhongshan School of Medicine, Sun Yat-Sen University, Guangzhou, China; ^2^ Department of Clinical Pharmacy, Guangzhou First People’s Hospital, School of Medicine, South China University of Technology, Guangzhou, China; ^3^ Program of Kidney and Cardiovascular Diseases, The Fifth Affiliated Hospital, Zhongshan School of Medicine, Sun Yat-Sen University, Guangzhou, China; ^4^ Department of Physiology, Pain Research Center, Zhongshan School of Medicine, Sun Yat-Sen University, Guangzhou, China; ^5^ Department of Cardiology, The Eighth Affiliated Hospital, Sun Yat-sen University, Shenzhen, China; ^6^ Guangdong Province Key Laboratory of Brain Function and Disease, Zhongshan School of Medicine, Sun Yat-Sen University, Guangzhou, China; ^7^ Key Laboratory of Cardiovascular Diseases, School of Basic Medical Sciences, Guangzhou Medical University, Guangzhou, China; ^8^ Program of Cardiovascular Research, The Eighth Affiliated Hospital, Zhongshan School Medicine, Sun Yat-sen University, Guangzhou, China

**Keywords:** ponatinib, adipose tissue macrophages, obesity, metabolic dysfunction, tyrosine kinase inhibitors

## Abstract

Obesity-induced metabolic syndrome is a rapidly growing conundrum, reaching epidemic proportions globally. Chronic inflammation in obese adipose tissue plays a key role in metabolic syndrome with a series of local and systemic effects such as inflammatory cell infiltration and inflammatory cytokine secretion. Adipose tissue macrophages (ATM), as one of the main regulators in this process, are particularly crucial for pharmacological studies on obesity-related metabolic syndrome. Ponatinib, a multi-targeted tyrosine kinase inhibitor originally used to treat leukemia, has recently been found to improve dyslipidemia and atherosclerosis, suggesting that it may have profound effect on metabolic syndrome, although the mechanisms underlying have not yet been revealed. Here we discovered that ponatinib significantly improved insulin sensitivity in leptin deficient obese mice. In addition to that, ponatinib treatment remarkably ameliorated high fat diet-induced hyperlipidemia and inhibited ectopic lipid deposition in the liver. Interestingly, although ponatinib did not reduce but increase the weight of white adipose tissue (WAT), it remarkably suppressed the inflammatory response in WAT and preserved its function. Mechanistically, we showed that ponatinib had no direct effect on hepatocyte or adipocyte but attenuated free fatty acid (FFA) induced macrophage transformation from pro-inflammatory to anti-inflammatory phenotype. Moreover, adipocytes co-cultured with FFA-treated macrophages exhibited insulin resistance, while pre-treat these macrophages with ponatinib can ameliorate this process. These results suggested that the beneficial effects of ponatinib on metabolic disorders are achieved by inhibiting the inflammatory phenotypic transformation of ATMs, thereby maintaining the physiological function of adipose tissue under excessive obesity. The data here not only revealed the novel therapeutic function of ponatinib, but also provided a theoretical basis for the application of multi-target tyrosine kinase inhibitors in metabolic diseases.

## Introduction

Obesity, especially visceral obesity, can lead to a series of systemic disorders such as hyperlipidemia, hyperglycemia and hypertension which are classified as metabolic syndromes (MS). Obesity-induced MS acts as one of the primary risk factors of coronary artery disease (CAD), stroke, cancer, non-alcoholic fatty liver disease (NAFLD) and type 2 diabetes mellitus (T2DM) and is therefore considered to be one of the major health problems in the modern world (Van Gaal et al., 2006). In dealing with obesity and the metabolic disorders, exercise and diet changes could bring great benefits, but it is difficult to persist in the long run. At present, a variety of pharmacological interventions are used in MS by increasing insulin sensitivity/secretion, regulating blood lipids and suppressing appetite. Although these drugs have proved to be beneficial for MS, there is still an urgent unmet need for more effective pharmacological intervention with less side-effect for obesity-related metabolic disorders (Kusminski et al., 2016).

Growing evidence has suggested that the dysfunction of white adipose tissue (WAT) is one of the earliest and most profound pathological changes in visceral obesity (Kahn et al., 2019). In the physiological state, adipose tissue plays a central role in regulating metabolic homeostasis by severing as a nutrient depository as well as an endocrine organ of multiple adipokines and cytokines. During obesity, dysfunctional WAT is accompanied by a shift in adipokines secretion profile, which typically shows a reduction in anti-inflammatory factors such as adiponectin and interleukin-10 (IL-10), while an elevation in pro-inflammatory cytokines such as TNFα, interleukin- 1β (IL-1β), IL-6 and IL-18. The impaired lipid storage function and abnormal inflammatory cytokine secretion of obese WAT together promote metabolic dysfunction (Tilg and Moschen, 2006; Burhans et al., 2018). In this process, adipose tissue macrophages (ATMs) play an indispensable role due to their high plasticity in response to the microenvironment changes (Kratz et al., 2014; McNelis and Olefsky, 2014). In the context of hypoxia and chronic inflammation, cytokines, chemokines and damage-associated molecular pattern (DAMP) such as free fatty acids (FFAs) released by adipocyte necrosis together promote the monocyte infiltration and pro-inflammatory M1 macrophages polarization. M1 macrophages surround apoptotic adipocytes to form a ‘crown-like' structure (CLS) (Cinti et al., 2005), which will lead to the overproduction of pro-inflammatory mediators and ultimately destroy the metabolic function of adipose tissue and the systematic insulin sensitivity. Therefore, the methods to block the obesity-related WAT inflammation and ATM activation would be beneficial for obesity and MS (Kusminski et al., 2016).

Ponatinib (Iclusig™; ARIAD Pharmaceuticals) is a multi-targeted tyrosine-kinase inhibitor approved by the US Food and Drug Administration (FDA) for patients with chronic myeloid leukemia and Philadelphia chromosome-positive acute lymphoblastic leukemia (O'Hare et al., 2009; Frankfurt and Licht, 2013). Interestingly, recent studies revealed that ponatinib can improve dyslipidemia and atherosclerotic plaque formation in apolipoprotein E-deficient (ApoE^−/−^) mice (Pouwer et al., 2018), suggesting that ponatinib may play a pivotal role in the regulation of metabolic disorders. However, the mechanisms to explain the effects of ponatinib on MS are not clear. Here, we observed that ponatinib is effective to ameliorate high-fat diet (HFD)-induced hyperlipidemia and NAFLD in leptin-deficient obesity mice model. Moreover, we provided evidence that inhibition of ATMs transformation from anti-inflammatory to pro-inflammatory phenotype underlies the beneficial effects of ponatinib on MS.

## Materials and methods

### Reagents

Ponatinib was purchased from Selleck (Houston, TX, United States). Antibodies against insulin receptor-beta; phospho-insulin receptor-beta (Tyr1150/1151); insulin receptor substrate-1; phospho-insulin receptor substrate-1 (Ser636/639); Akt; phosphor-Akt (Ser 473) were purchased from Cell Signaling Technology (Danvers, MA, United States). Antibody against F4/80 was obtained from Servicebio Biotechnology (Wuhan, China). Alexa Fluor®-700 anti-mouse CD38 antibody was obtained from eBioscience (San Diego, CA, United States). Isobutyl-methyl-xanthine (IBMX), dexamethasone, palmitic acid, oleic acid and Oil Red-O were obtained from Sigma-Aldrich (St. Louis, MO, United States). Culture medium, and fetal bovine serum (FBS) were obtained from Gibco (Carlsbad, CA, United States). Insulin was purchased from Novo Nordisk (Copenhagen, Denmark). Macrophage colony-stimulating factor 1 (M-CSF1) was purchased from Novoprotein Scientific (Shanghai, China). Sodium Oleate, Sodium palmitate were purchased from Sigma-Aldrich (St. Louis, MO, United States). DNA ladder, Nucleic Acid Dye and agarose were purchased from Genstar (Beijing, China).

### Animal study

All animal experimental procedures were performed in accordance with the policy of the Sun Yat-Sen University Animal Care and Use Committee and conformed to the Guidelines for the ethical review of laboratory animal welfare People’s Republic of China National Standard GB/T 35,892-2018. Mice were housed under specific pathogen-free conditions with 12/12 h light/dark cycle and had *ad libitum* access to water and food. Different groups were allocated in a randomized manner and investigators were blinded to the allocation of different groups when doing outcome evaluations. Sample size was determined by pilot experiments and exact n-numbers were shown in the respective figure legend. All mouse tissue samples used in this study were collected after euthanasia by intraperitoneal injection of an overdose of sodium pentobarbital (200 mg/kg).

6-week-old male ob/ob mice and its lean littermates with a genetic background of C57BL/6J were procured from Cyagen Biosciences (Santa Clara, CA, United States). After 2 weeks of adaptation, the ob/ob mice and lean mice were divided into four groups randomly with five animals per group. The drug intervention groups (lean + ponatinib and ob/ob + ponatinib) were treated with ponatinib (5 mg/kg made in 25 mM citrate buffer (pH 2.75)] *via* intragastric administration for 8 weeks, while the control groups were treated with vehicle (citrate buffer). Body weight was measured weekly. After treatment for 8 weeks, fasting blood glucose levels, random blood glucose levels, oral glucose tolerance test (GTT) and insulin tolerance test (ITT) were measured with glucometer. At the end of the experiment, the mice after an overnight fast were euthanized and the blood, liver and adipose tissue samples were collected for subsequent testing.

### Glucose and insulin tolerance test

GTT and ITT were performed as previously described (Guo et al., 2020). In brief, GTT and ITT were examined in mice fasted for 12 and 6 h, respectively. Mice were intraperitoneal administration with 1 g/kg glucose (made in 0.9% NaCl) for GTT, while 0.75 U/kg human insulin (made in 0.9% NaCl) was intraperitoneally injected into mice for ITT. Blood glucose were monitored with a glucometer at 0, 15, 30, 60, 120 min. The blood glucose change over time curve was drawn and calculate the area (AUC) under the curve using the GraphPad Prism 8.0 (GraphPad Software, La Jolla, CA, United States).

### Histology and immunohistochemistry staining

Four% paraformaldehyde-fixed paraffin-embedded murine tissues were subjected to hematoxylin and eosin (H&E) staining to observe the histological changes in the tissues. Liver sections were embedded in OTC, sliced (7 μm) under freezing conditions and applied for Oil Red-O staining for assessment of lipid deposition.

For immunohistochemistry staining, paraffin sections were deparaffinized, rehydrated, antigen retrieved by immersed in 0.01 M citrate buffer, blocked and then incubated with anti-F4/80 at 1:1000 overnight at 4°C. After washed three times by PBS, sections were tested using Real Envision Detection kit (Dako, Glostrup, Denmark) according to the manufacturer’s instructions.

### Biochemistry analysis and hepatic lipid analyses

Blood was collected from the Retro-orbital bleeding under terminal anesthesia. Samples were centrifuged at 1000 x *g* for 20 min and then supernatants were collected. Insulin in serum was performed by enzyme-linked immunosorbent assay (ELISA) (RayBiotech, Norcross, GA, United States). Serum lipids, including serum total cholesterol (TC), triglyceride (TG), high-density lipoprotein cholesterol (HDL-C) and low-density lipoprotein cholesterol (LDL-C), were detected by analytical auto analyzer (Mindray, Shenzhen, China). Commercial kits (Applygen, Beijing, China) were used to measure liver tissue triglyceride (TG) and total cholesterol (TC) according to the manufacturer’s protocol.

### Cell culture and cell differentiation

Human normal hepatocyte LO2 and mouse embryonic fibroblast 3T3-L1 were purchased from Procell Life Science and Technology Co. Ltd. All cell lines were maintained in standard medium containing Dulbecco’s modified Eagle’s medium (DMEM), 10% fetal bovine serum (FBS),1% penicillin–streptomycin at 37°C in 5% CO2.

3T3-L1 adipocytes was differentiated as previously described (Qi et al., 2019). 3T3-L1 preadipocytes were cultured to confluence (day 0) and disposed in differentiating medium containing Isobutyl-methyl-xanthine (IBMX) (0.5 mmol/L), dexamethasone (1 μmol/L) and insulin (10 μg/ml) for 2 days. The medium was renewed to differentiation-maintenance medium comprising 10 μg/ml insulin for 2 days. Thereafter, the medium was changed to a standard medium and replaced every 2 days. At day 8–12, mature adipocytes can be identified by 60% Oil Red-O solution or used in subsequent experiments.

For lipid droplet deposition experiment in LO2, cells were treated with FFA mixture for 24 h. Afterward, lipid droplet located in these cells were stained with Oil Red-O solution as previously described (Guo et al., 2020).

BMDMs isolation and differentiation were performed as previously described (Pineda-Torra et al., 2015). In short, 8-week-old mice were anesthetized and sacrificed. The abdomen and hind legs of mice were sterilized with 75% ethanol. The mice were cut an incision in the middle line of the abdomen, exposed the hind legs, and removed all muscle tissue from the bone using scissors. The femur and tibia were separated at the knee joint of the mouse. Bone marrow was then flushed by the medium (RPMI1640). The bone marrow cells were pipetted up and down to become single—cell suspension. The cells were centrifuged at 500 x *g* for 5 min and then supernatants were removed. The sediment was resuspended using a complete medium containing macrophage colony stimulating factor-1 (M-CSF1) (20 ng/ml) and the cells were plated as needed and cultured at 37°C in 5% CO_2_. Fresh complete medium was replaced every 3 days and non-adherent cells were removed. After 7 days, fully differentiated macrophages were obtained.

### Co-culture of 3T3 and bone marrow-derived macrophages cells

3T3-L1 adipocytes and BMDMs were seeded into 6-well plates respectively and differentiated according to the method in the previous section. On the eighth day, the matured BMDMs was digested and transferred into polycarbonate membrane inserts (Corning, NY, United States), and treated with corresponding reagents such as ponatinib and FFA for 48 h. On the 10th day, the polycarbonate membrane inserts with treated BMDMs were transferred into 6-well plates and co-cultured with mature 3T3 adipocytes. After 24 h, the lysate of 3T3 adipocytes was collected and proceeded.

### Preparation of free fatty acid mixture solution

A mixture solution of FFA was prepared as previously described (Lim et al., 2017). Firstly, Sodium Oleate and Sodium Palmitate were prepared as 10 mM stock solutions separately. Subsequently, to prepare the working solution of the FFA mixture, the Palmitate, Oleate and fatty-acid-free bovine serum albumin (BSA) solutions were mixed and diluted with cell culture medium, and then the pH value was adjusted to 7.1 with hydrochloric acid. The final concentration of each component in the FFA solution was 0.66 mM Oleate, 0.33 mM Palmitate and 1% BSA. Accordingly, 1% BSA solution was used as blank control.

### Quantitative real time-PCR analysis

To determine mRNA levels, total RNA was isolated from liver tissue, adipose tissue, 3T3-L1 or BMDM cells by using TRIzol reagent according to manufacturer’s instructions. RNA (2 μg) was reverse transcribed into cDNA using the QuantiTect Reverse Transcription Kit (Qiagen, Hilden, Germany). Real-time PCR was performed using SYBR Green PCR Master Mix (Invitrogen, Carlsbad, CA, United States) on a CFX96 Real-Time PCR Detection System (Bio-Rad, Hercules, CA, United States) or a LightCycle480 II Real-Time PCR Detection System (Roche, Basel, Switzerland). The sequence-specific primers for the genes are listed in Supplementary Table S1. The fold change in expression of each gene was calculated using the 2^−ΔΔCT^ method, with glyceraldehyde-3-phosphate dehydrogenase as an internal control.

### Agarose gel electrophoresis

The PCR-amplified products were electrophoretically separated on 3% agarose gel in Tris–acetate–EDTA (TAE) buffer (1×). Agarose gels were stained with Nucleic Acid Dye and visualized under UV light from the Gel Record System (ChemiDoc XRS+, Bio-Rad, Hercules, CA, United States).

### Flow cytometry analysis

Flow cytometry analysis was performed as previously described (Jablonski et al., 2015). We use FFA mixture to stimulate differentiated and mature macrophages for 24 h, and then use trypsin to prepare a single cell suspension for fluorescence-activated cell sorting (FACS) staining. After centrifuged at 500x *g* for 5 min the supernatants were removed, then the cells were resuspended with 50 μL of Fc receptor blocker and incubate at room temperature for 20 min. Afterward, cells were incubated with anti-mouse CD38 antibody at room temperature for 20 min. After the antibody incubation, the cells were diluted with 400 μL PBS and analyzed on a flow cytometer (CytoFLEX-S, Beckman Coulter, Inc., CA, United States) according to the manufacturer’s instructions.

### Western blot

Western blotting was performed as previously described (Huang et al., 2014). Briefly, cells were lysed in lysis buffer which were added with phosphatase inhibitors and protease. Proteins were quantified by the Bradford assay (Thermo Fisher Scientific, Waltham, MA, United States). The proteins were fractionated on a 10%SDS-PAGE gel and then transferred onto polyvinylidene difluoride membranes. The membranes were incubated with different primary antibodies overnight at 4°C and subsequently incubated with Peroxidase-conjugated secondary antibodies. Enhanced chemiluminescence reagents (Merck Millipore, Darmstadt, Germany) were used to visualize the blots according to manufacturer’s protocol. ImageJ4.1 software was used to quantify the immunoreactive bands.

### Statistical analyses

All data are expressed as the mean ± standard error of the mean (SEM). Statistical analysis was performed using SPSS 16.0 software (SPSS Inc., Chicago, IL, United States) and unpaired two-tailed Student’s t-test or one-way analysis of variance (ANOVA) followed by Bonferroni’s multiple comparisons post hoc test with a 95% confidence interval. Correlation analyses were performed using the Pearson correlation test. *p* < 0.05 was considered statistically significant.

## Results

### Ponatinib improved insulin sensitivity in obese mice

**FIGURE 1:**
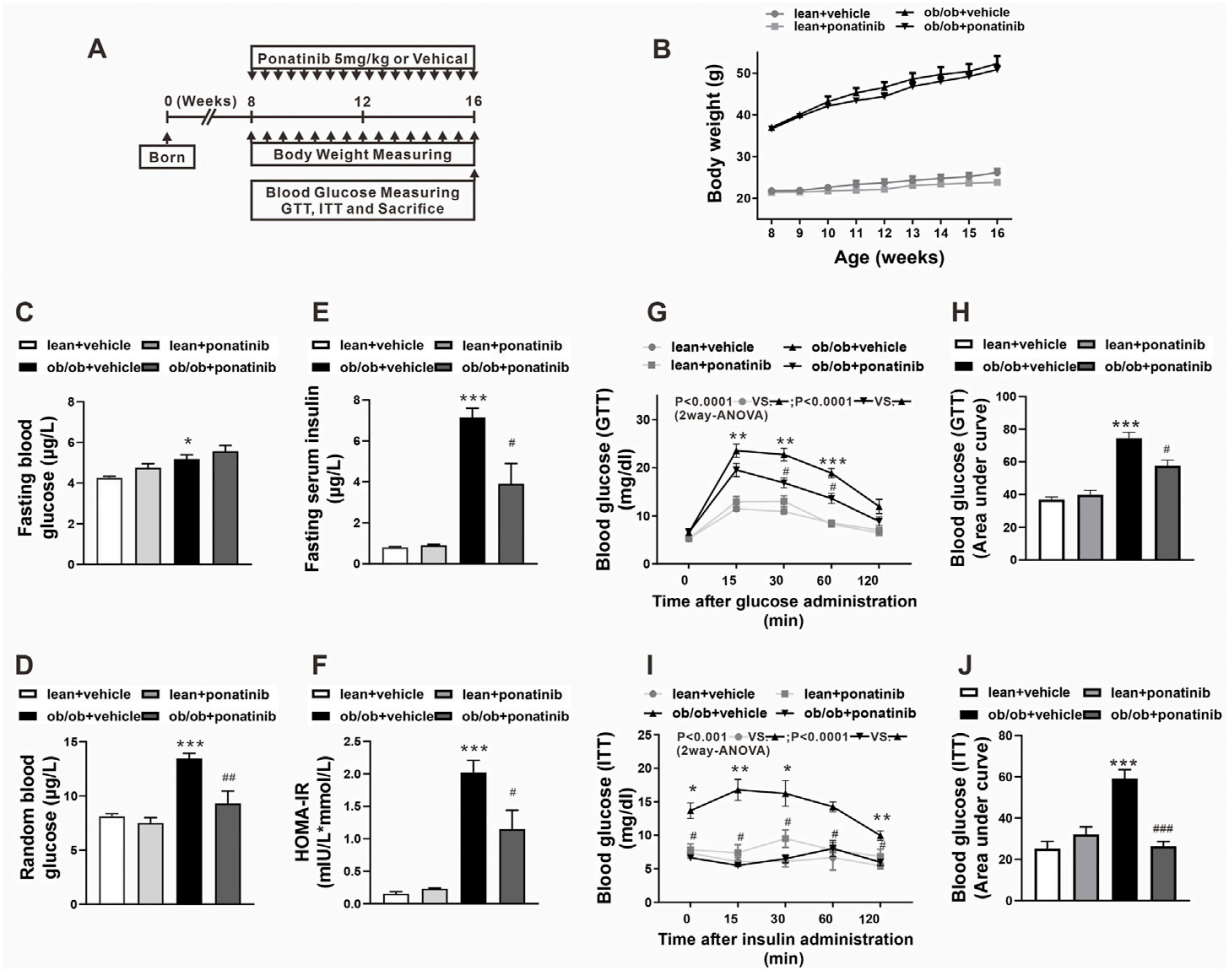
Ponatinib improved insulin resistance in ob/ob mice **(A)** Schematic diagram of experimental protocol for studying the effect of ponatinib in ob/ob mice and lean mice. **(B)** Body weight of ob/ob mice and the lean mice after administration with or without ponatinib. *n* = 5. **(C–F)** Fasting blood glucose **(C)**, random blood glucose **(D)**, fasting serum insulin **(E)** and HOMA-IR index **(F)** of ob/ob and lean mice with or without ponatinib. *n* = 5. **(G–J)** GTT **(G)** and ITT **(I)** performed on ob/ob mice and lean mice after 8-week administration with or without ponatinib. Blood glucose area under the curve **(H–J)** measured from G and I. *n* = 5. Statistical comparisons were performed with RM two-way ANOVA with Bonferroni’s multiple comparisons test. **(G–I)** and one-way ANOVA **(B–J)** followed by Bonferroni’s multiple comparisons post hoc test. Data represent mean ± SEM. *p < 0.05, **p < 0.01*,* ***p < 0.001 *vs.* lean + vehicle. #*p* < 0.05, ##*p* < 0.01*,* ###*p* < 0.001 vs*.* ob/ob + vehicle.

To clarify the potential effect of ponatinib on metabolic disorders during obesity, ponatinib was orally administered to leptin-deficient (ob/ob) mice and the control mice (lean) once-daily for 8 weeks (Figure 1A). As expected, body weight and weight gain of ob/ob mice were significantly higher than those of lean mice, but ponatinib treatment had no effect on body weight in both two groups (Figure 1B). Both the fasting and the random blood glucose levels in ob/ob mice were increased compared with the lean mice at 16 weeks (Figures 1C,D). Ponatinib did not affect the fasting glucose level in ob/ob mice and control mice (Figure 1C). However, the random blood glucose level of ob/ob mice was significantly reduced after ponatinib administration (Figure 1D). Evidently, ob/ob mice at 16 weeks have higher fasting serum insulin level and homeostasis model assessment of insulin resistance (HOMA-IR) value compared with lean mice, ponatinib treatment partially restored these abnormal increases. (Figures 1E,F). Moreover, ponatinib ameliorated glucose tolerance and insulin sensitivity in ob/ob mice as determined by intraperitoneal glucose tolerance test (GTT) and insulin tolerance test (ITT) (Figures 1G–J).

### Ponatinib reduced serum lipid levels and hepatic lipid deposition in ob/ob mice

**FIGURE 2:**
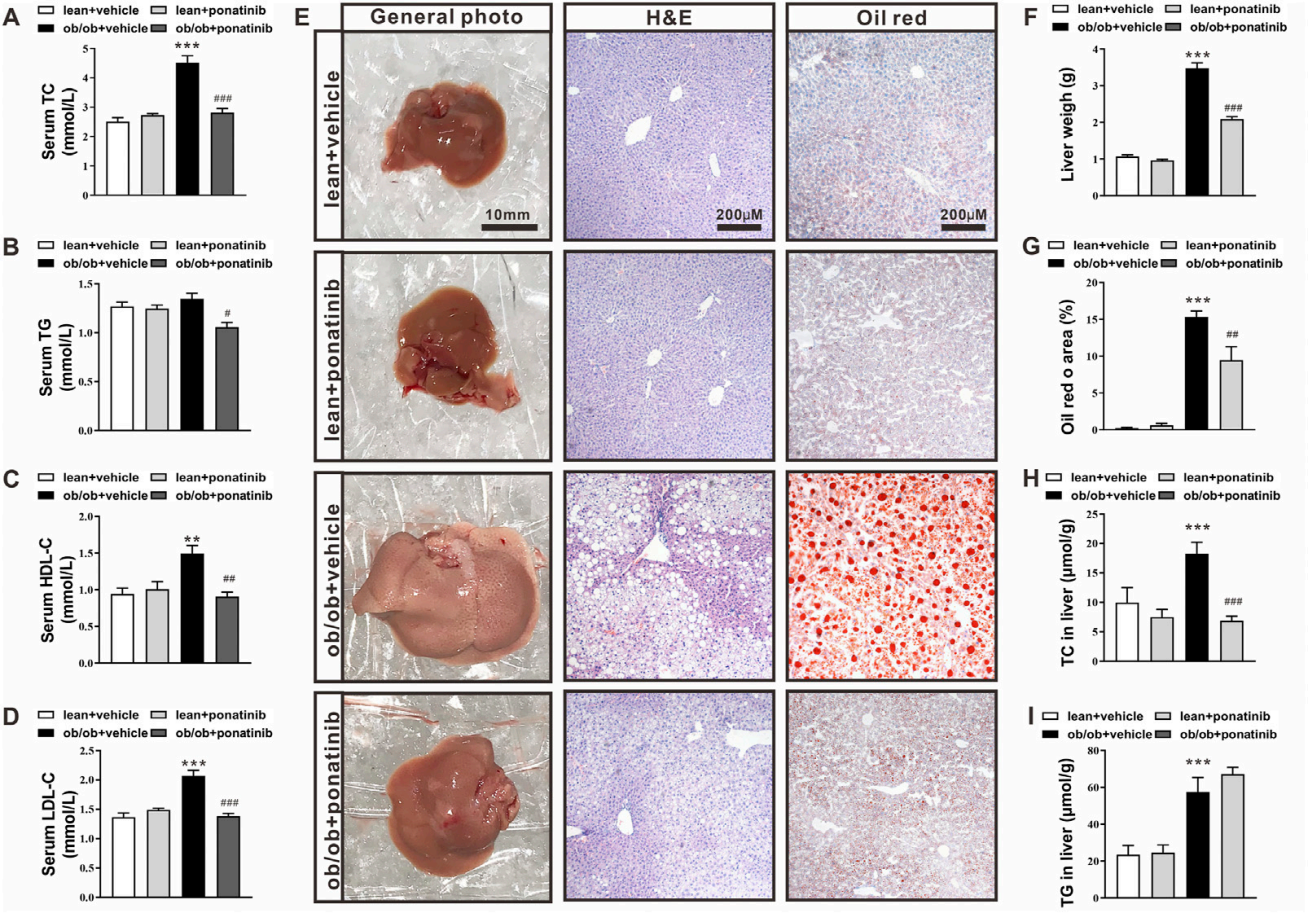
Ponatinib improved lipid ectopic deposition in ob/ob mice **(A–D)** Serum TC **(A)**, TG **(B)**, HDL **(C)**, LDL **(D)** of ob/ob mice and lean mice treated with or without ponatinib. *n* = 5. **(E)** Representative images of liver, HE and Oil Red O staining of livers tissues from ob/ob mice and lean mice with or without ponatinib. *n* = 5. Scale bar,10 mm, 200 μm. **(F)** Liver weight in ob/ob mice and control mice treated with ponatinib or vehicle. *n* = 5. **(G)** Quantification of oil red area from **(E) (H–I)** Liver TC **(H)**, TG **(I)** levels in ob/ob mice or lean mice administrated with ponatinib or vehicle. *n* = 5. Statistical comparisons were performed with one-way ANOVA **(A–D) (F–I)** followed by Bonferroni’s multiple comparisons post hoc test. Data represent mean ± SEM. **p < 0.01*,* ***p < 0.001 vs*.* lean + vehicle. #*p* < 0.05, ##*p* < 0.01*,* ###*p* < 0.001 vs*.* ob/ob + vehicle.

At 16 weeks of age, ob/ob mice exhibited higher levels of serum total cholesterol (TC), high density lipid cholesterol (HDL-C), and low density lipid cholesterol (LDL-C) compared with lean mice. Ponatinib intervention significantly reduced serum TG, TC, HDL-C, and LDL-C in ob/ob mice (Figures 2A–D). Histological analysis using HE and Oil Red-O staining revealed greater lipid accumulation in livers of ob/ob mice than lean mice (Figure 2E). Consistently, ob/ob mice exhibited increased liver weight (Figure 2F) and elevated TG and TC levels in the liver (Figures 2F–I). Ponatinib treatment obviously reduced liver lipid accumulation, liver weight, and lowered liver TC levels of ob/ob mice (Figures 2E–H) However, ponatinib had no effect on liver TG levels of ob/ob mice (Figure 2I).

### Ponatinib protects the function of obese adipose tissue and suppresses inflammation

**FIGURE 3:**
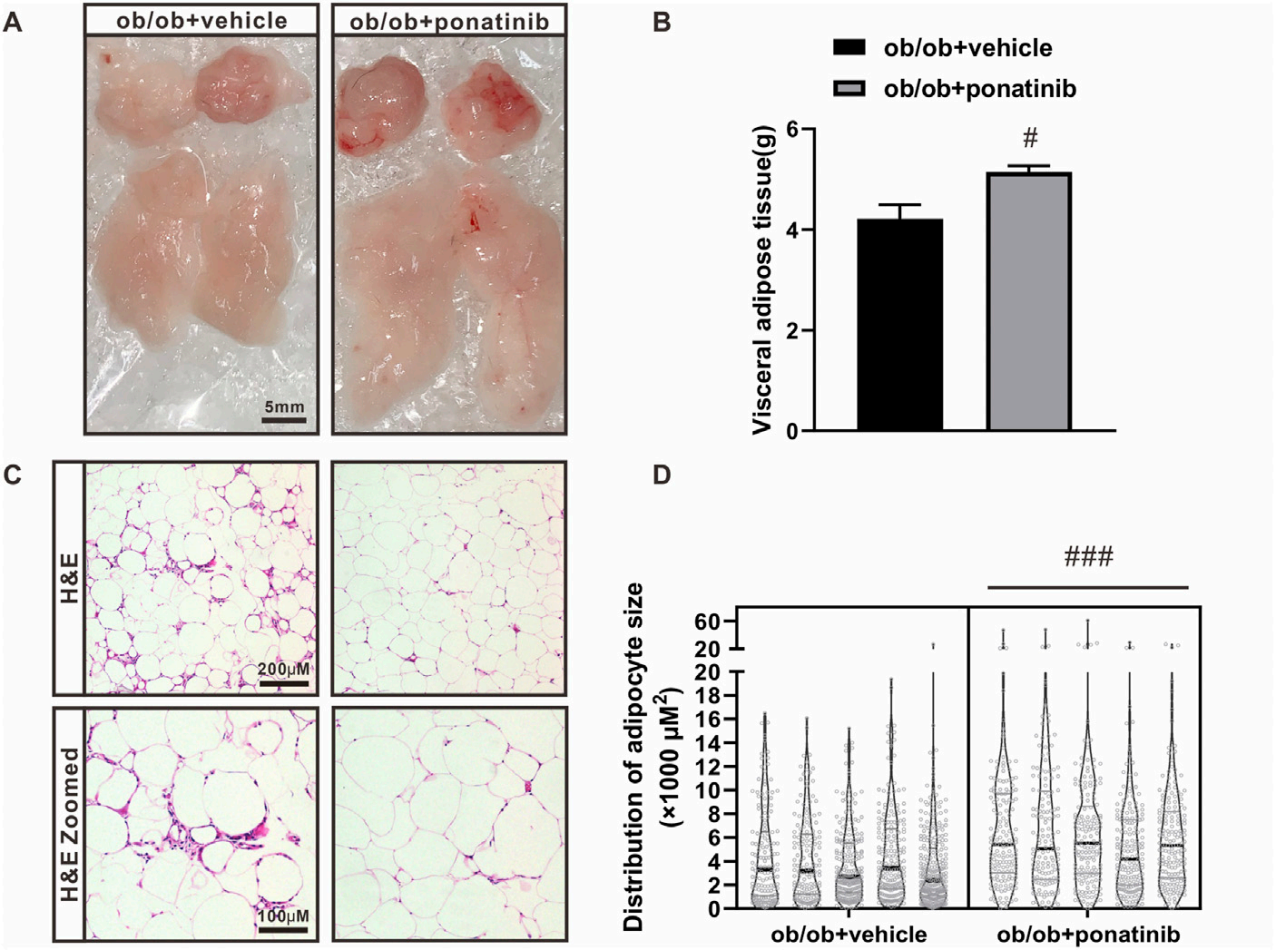
The lipid storage function of adipose tissue in ob/ob mice was retained by ponatinib **(A)** The visceral white adipose tissue of ob/ob mice in ponatinib group or vehicle group. *n* = 5. Scale bar, 5 mm. **(B)** Weight of visceral white adipose tissue of ob/ob mice treated with or without ponatinib. *n* = 5. **(C)** Representative images of HE staining of visceral white adipose tissue of ob/ob mice in ponatinib group or vehicle group. *n* = 5. Scale bar, 100 μm and 200 μm. **(D)** Analyzation of adipocyte size in **(C)** *n* = 5. Statistical comparisons were performed with unpaired two-tailed Student’s t-test **(B–D)**. Data represent mean ± SEM. ^#^ *p* < 0.05, ^###^ *p* < 0.001*vs.* ob/ob + vehicle.

**FIGURE 4:**
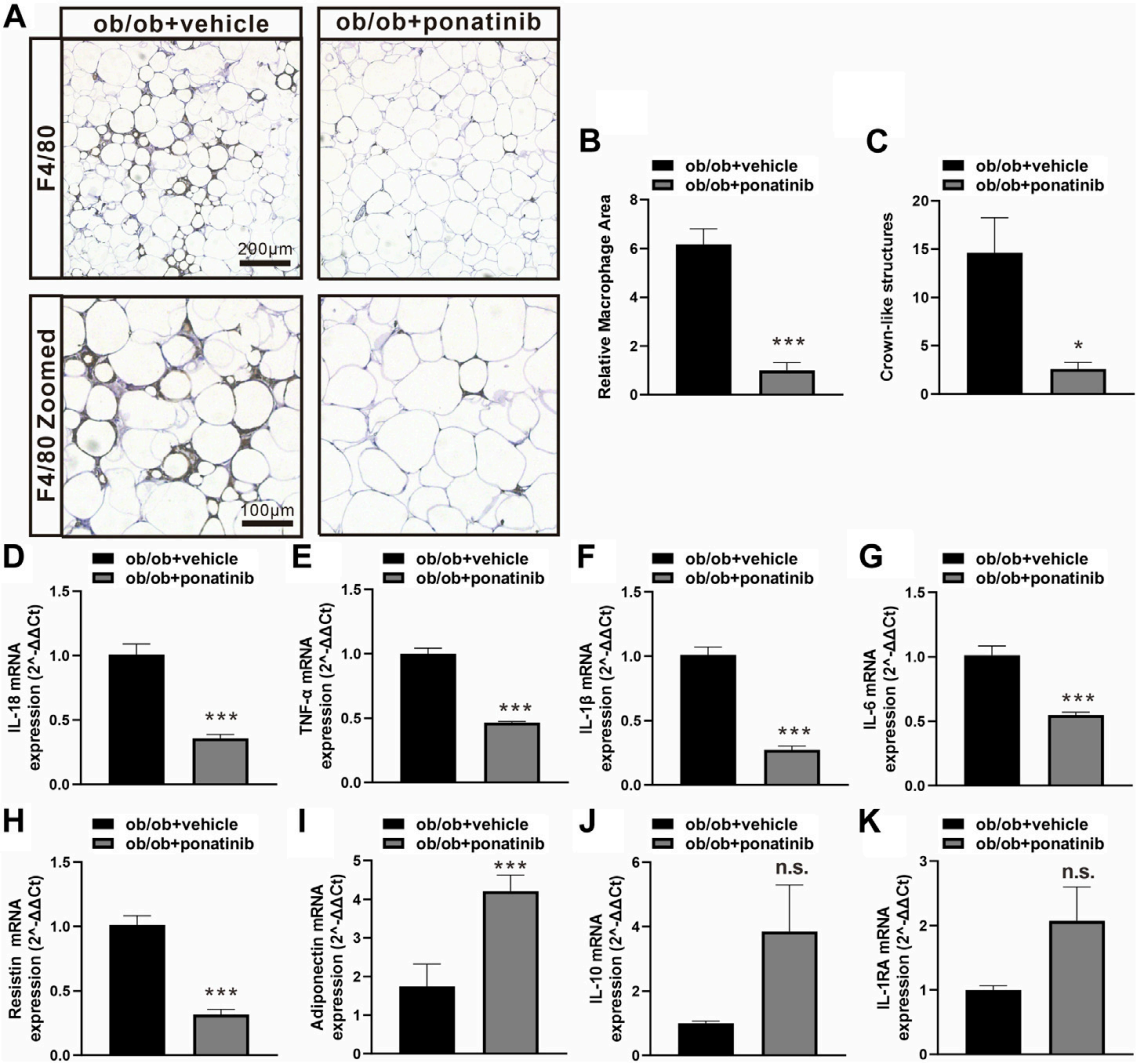
Ponatinib inhibited macrophage infiltration and cytokine expression in adipose tissue **(A)** Representative image of F4/80 immunostaining of visceral adipose sections of ob/ob mice in ponatinib group and control group. *n* = 5. Scale bar, 100 μm and 200 μm **(B)** Quantification of relative macrophage from **(A)** *n* = 5 **(C)** Quantitative analysis of crown-like structures from **(A)** *n* = 5. **(D–K)** mRNA levels of gene associated with inflammatory response in visceral adipose tissue of ob/ob mice treated with ponatinib or vehicle. *n* = 4. Statistical comparisons were performed with unpaired two-tailed Student’s t-test **(B–K)**. Data represent mean ± SEM. n.s. *p* > 0.05, ^*^ *p* < 0.05, ^***^ *p* < 0.001 vs. ob/ob + vehicle.

**FIGURE 5:**
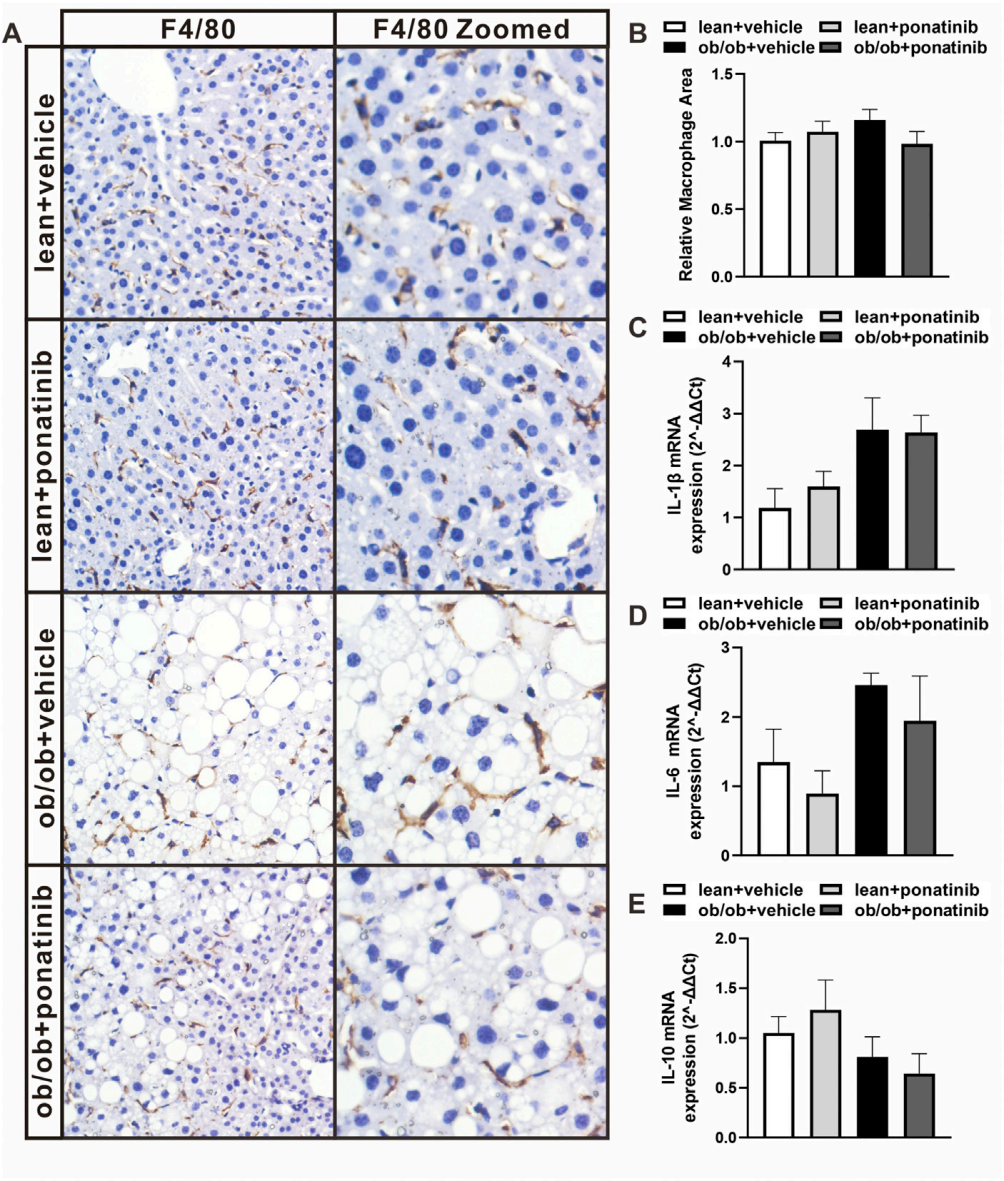
Ob/ob mice and lean mice had similar macrophage infiltration and inflammatory factor levels in liver with or without ponatinib. **(A)** Representative image of F4/80 immunostaining of liver sections of ob/ob mice and lean mice administrated with ponatinib or vehicle. *n* = 5. **(B)** Relative macrophage area of each group from **(A)** n = 5. **(C–E)** IL-1β, IL-6, and IL-10 mRNA transcription from liver of ob/ob mice and lean mice treated with or without ponatinib measured by qPCR. *n* = 5. Statistical comparisons were performed with one-way ANOVA **(B–D)** followed by Bonferroni’s multiple comparisons post hoc test. Data represent mean ± SEM.

Although the liver weight of ob/ob mice in ponatinib group was significantly lower than that in vehicle group, we surprisingly found that the visceral white adipose tissue (WAT) weight of ob/ob mice after ponatinib treatment was not reduced but increased compared to the vehicle group (Figures 3A,B). Thus, we assessed the morphology of these WAT by HE staining (Figure 3C). We found that the size of adipocytes in ponatinib treated group tended to be larger than that in the vehicle group (Figure 3D). Studies have shown that the size distribution of adipocytes in obese individuals, especially the increase of small adipocytes, is not only highly correlated with type 2 diabetes, but also reflects the dysfunctions in adipose storage and inflammation (McLaughlin et al., 2007; McLaughlin et al., 2010; Fang et al., 2015). To this end, we investigated the infiltration of macrophages and the severity of inflammation in obese WAT of this model. We observed that the intervention of ponatinib could not only significantly reduce the infiltration of macrophages in obese WAT, but also reduce the amount of “crown like” structure (Figures 4A–C). Consistently, several pro-inflammatory cytokines such as IL-1β, TNF-α, IL-18, IL-6, and resistin were remarkably decreased after ponatinib treatment (Figures 4D–H). Moreover, ponatinib also significantly increased the level of adiponectin in obese WAT (Figure 4I), but there were no significant differences in other anti-inflammatory cytokines such as IL-10 and IL-1RA (Figures 4J,K). There was no change in macrophage infiltration and inflammatory cytokine levels in the liver of ob/ob mice after ponatinib treatment (Figures 5A–E).

### Ponatinib had no effect on the metabolic phenotype of hepatocytes and adipocytes

**FIGURE 6:**
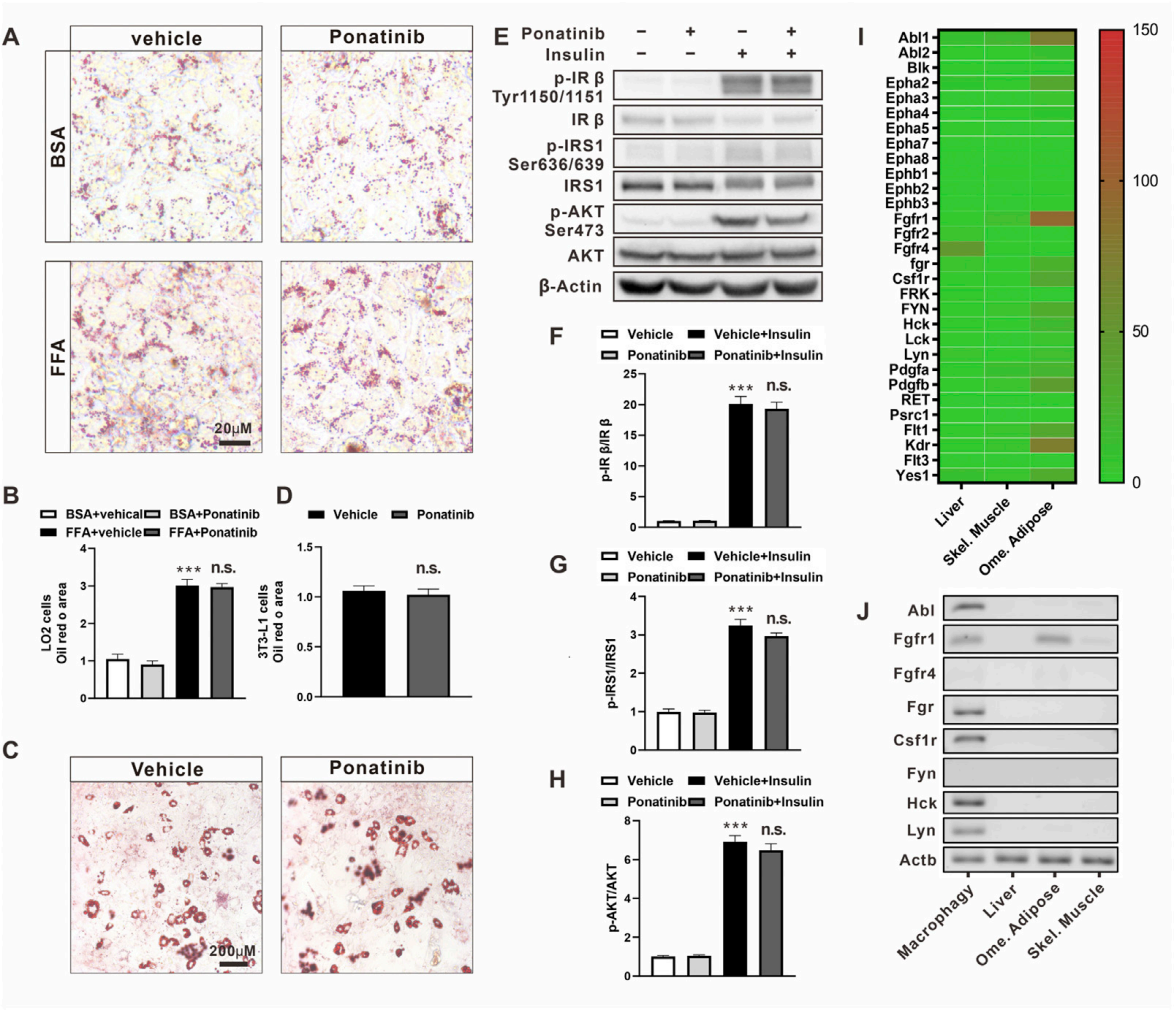
The metabolic profile of hepatocytes and adipocytes was not directly affected by ponatinib. **(A)** Representative Oil Red O staining of LO2 cells administrated with ponatinib or vehicle after FFA or BSA stimulation for 24 h *n* = 6. Scale bar, 20 μm **(B)** Quantitative analysis of oil red area of LO2 cells in **(A)**. Statistical comparisons were performed with one-way ANOVA followed by Bonferroni’s multiple comparisons post hoc test. Data represent mean ± SEM, ***p < 0.001 vs. BSA + vehical; n. s. *p* > 0.05 *vs.* FFA + Vehicle. **(C)** Representative Oil Red O staining of 3T3-L1 adipocytes treated with ponatinib or vehicle. *n* = 6. Scale bar, 200 μm **(D)** Quantitative analysis of oil red area of 3T3-L1 adipocytes in **(C)**. *n* = 6. Statistical comparisons were performed with unpaired two-tailed Student’s t-test, Data represent mean ± SEM, n. s. *p* > 0.05 vs*.* Vehicle. **(E)** Representative western blot showing levels of total and phosphorylation of IR-β (Tyr1150/1151), IRS1(Ser636/639), AKT (Ser473) of 3T3-L1 adipocytes in response to insulin stimulation for 30 min with or without ponatinib treatment. *n* = 4. **(F–H)** Quantification of phosphorylation of IR-β (Tyr1150/1151), IRS1 (Ser636/639), AKT (Ser473) expression level in **(E)**. *n* = 4. Statistical comparisons were performed with one-way ANOVA followed by Bonferroni’s multiple comparisons post hoc test. Data represent mean ± SEM, ***p < 0.001 vs. Vehical; n. s. *p* > 0.05 vs. Vehicle + Insulin. **(I)** The expression profile of ponatinib’s high-affinity targets in liver, skeletal muscle and omentum adipose from Genotype-Tissue Expression (GTEx) Project. Color scale indicates mean TPM (transcripts per million) value. **(J)** Representative electropherogram of one of six mice qPCR products to verify FGR, HCK, Lyn, CSFR, Fyn, FGFR, and ABL mRNA expression levels in BMDM, visceral fat, liver, and skeletal muscle.

In order to further study the specific regulation of ponatinib on obesity-related metabolic disorders, we conducted a series of *in vitro* experiments. Firstly, we exposed human fetal hepatocyte line, LO2 (Hu et al., 2013), to a mixture of free fatty acids (palmitic acid, oleic acid and BSA) for 24 h to induce lipid drop disposition. By Oil Red-O staining, we observed that there is a higher degree of lipid droplet deposition under culturing with FFA compared to BSA, but ponatinib had no significant effect on the lipid deposition in these cells (Figures 6A,B). Secondly, we used the classic IBMX-Dex-insulin triple induction method to drive the differentiation of preadipocyte line, 3T3-L1, into adipocytes. However, we did not observe any significant change in the differentiation ratio of adipocytes by ponatinib (Figures 6C,D). Finally, we examined the effect of ponatinib on insulin sensitivity of 3T3-L1 adipocytes. Western blot results showed that ponatinib did not change the phosphorylation levels of insulin receptor-beta (p-IRβ, Tyr1150/1151), insulin receptor substrate-1 (IRS1, Ser636/639) and Akt (Ser473) in adipocytes under the stimulation of insulin (Figures 6E–H).

These results showed that the effects of ponatinib on the metabolic properties of ob/ob mice cannot be reproduced in the individual hepatocyte or adipocyte cell lines *in vitro*. We then analyzed the expression profile from the Genotype-Tissue Expression Project (GTEx, version8) to check the expression profile of ponatinib targets in WAT, liver and skeletal muscle (Lonsdale et al., 2013). As the data showed, about one-third of the 30 major targets of ponatinib were expressed in adipose tissue, but not in liver or skeletal muscle (Figure 6I). Given that there is a range of ponatinib targets expressed in adipose tissue, we were wondering why does ponatinib have no effect on insulin sensitivity in adipocytes cultured *in vitro*? Considering that macrophage is a crucial type of cell in regulating adipose tissue function, we next isolated mouse bone marrow-derived macrophages (BMDM) and compared its expression of ponatinib targets with adipose, liver and skeletal muscle tissue by RT-PCR. Consistent with the previous study (Lowell, 2004), we confirmed the expression of Abl1, Fgr, Hck, Lyn, and Csf1r gene in BMDM; Furthermore, except of Fgfr1, which was expressed in adipose tissue, the rest of these targets could hardly be detect in adipose, liver or skeletal muscle tissues by RT-PCR, i.e., CT value ≥ 35. (Figure 6J; Supplementary Table S2).

### Ponatinib blocked free fatty acid-induced inflammatory activation of macrophages

**FIGURE 7:**
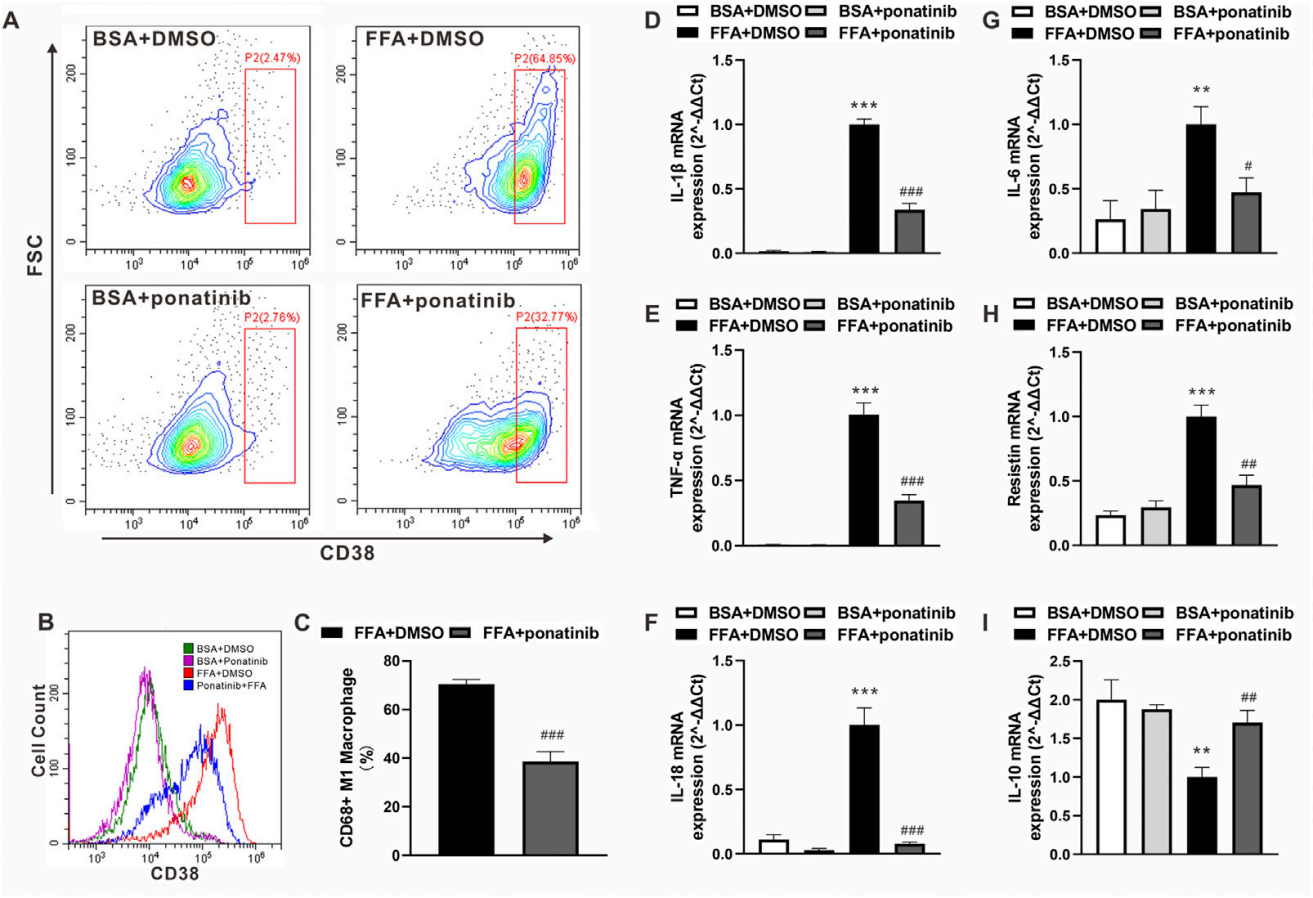
Ponatinib attenuated FFA induced Inflammatory phenotypic transformation of macrophages. **(A)** Flow cytometry analysis of the distribution of CD38 in BMDM cultured with FFA or BSA after treated with ponatinib or vehicle. **(B)** Representative overlayed histogram in **(A) (C)** Analysis of CD38 positive M1 macrophage ratio after FFA treatment in **(A)** *n* = 6. **(D–I)** mRNA levels of gene associated with inflammatory response in FFA treated BMDMs with or without ponatinib. *n* = 4. Statistical comparisons were performed with unpaired two-tailed Student’s t-test **(C)** or one-way ANOVA **(D–I)** followed by Bonferroni’s multiple comparisons post hoc test. Data represent mean ± SEM, **p < 0.01*,* ***p < 0.001 *vs.* BSA + DMSO. ^#^ *p* < 0.05, ^##^ *p* < 0.01*,* ^###^ *p* < 0.001 vs.FFA + DMSO.

In view of the expression pattern of ponatinib’s high affinity targets in macrophages, we speculated whether the inflammatory activation of ATMs in obesity would be regulated by ponatinib. FFA, one of the major damages associated molecular patterns (DAMP) released by apoptotic adipocytes in obese adipose tissue, acts as a crucial stimulus in the inflammatory phenotype transformation of ATMs. Therefore, we analyzed the distribution of CD38, a murine M1 macrophage marker (Jablonski et al., 2015), in BMDM cultured with FFA. As showed by flow cytometry analysis, most of the naive BMDMs transformed into CD38 positive M1 macrophages under FFA stimulation, while the expression intensity of CD38 and the proportion of M1 macrophages decreased significantly under the intervention of ponatinib (Figures 7A–C). Furthermore, we analyzed the expression of inflammatory factors in FFA treated BMDMs. QPCR results showed that the expression levels of IL-1β, TNF-α, IL-18, IL-6 and resistin in FFA treated group were significantly higher than those in BSA group, ponatinib treatment significantly blocked the effects of FFA (Figures 7D–H). In addition, ponatinib also reversed the inhibition of IL-10 by FFA (Figure 7I).

### Ponatinib protected insulin sensitivity of adipocytes co-cultured with macrophages

**FIGURE 8:**
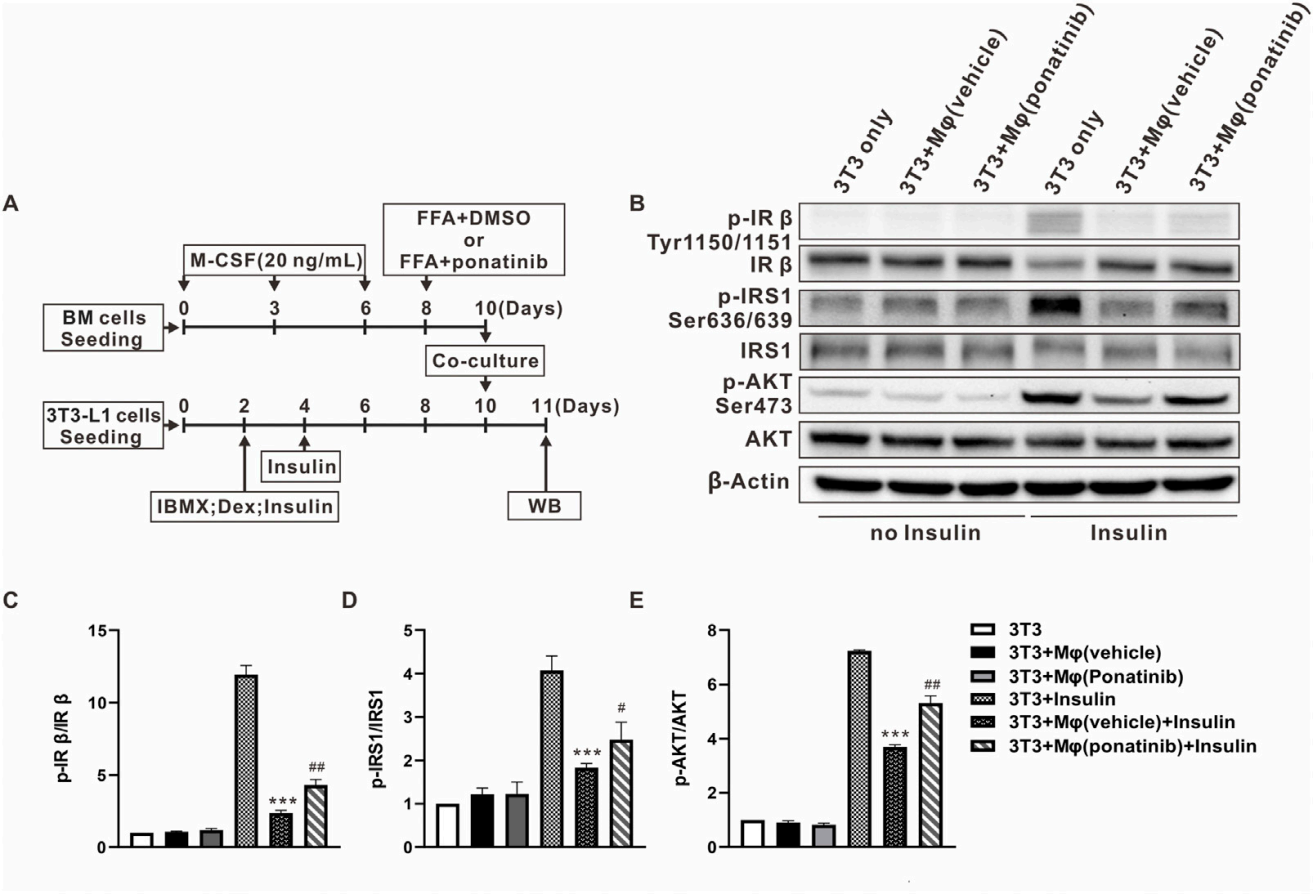
Insulin sensitivity of adipocytes co-cultured with macrophages was protected by ponatinib. **(A)** Schematic diagram of experimental design for studying the effect of ponatinib in insulin sensitivity of adipocytes co-cultured with macrophages. **(B)** Representative western blot showing levels of total and phosphorylation of IR-β (Tyr1150/1151), IRS1(Ser636/639), AKT (Ser473) of adipocytes co-cultured with macrophages in response to insulin for 30 min with or without ponatinib treatment. *n* = 4. **(C–E)** Quantification of phosphorylation of IR-β (Tyr1150/1151), IRS1(Ser636/639), AKT (Ser473) expression level in **(B)** *n* = 4. Statistical comparisons were carried out with one-way ANOVA **(C–E)** followed by Bonferroni’s multiple comparisons post hoc test. Data represent mean ± SEM, ***p < 0.001 vs. 3T3+Insulin. ^#^ *p* < 0.05, ^##^ *p* < 0.01 vs. 3T3+Mφ(vehicle)+Insulin.

As ponatinib hinders the FFA-induced inflammatory phenotype transformation of macrophages, we are curious whether ponatinib can improve the insulin sensitivity of adipose tissue through this function. So we then designed the following co-culture experiments of BMDMs and 3T3-L1 cells (Figure 8A). On one hand, we used FFA to induce the inflammatory phenotype of BMDMs on the eighth day of differentiation, with or without ponatinib. On the other, 3T3-L1 cells were used to differentiate into adipocyte. These two types of cells were then co-cultured at the 10th day for 24 h, and the insulin sensitivity of 3T3-L1 adipocytes was assessed by western blot. Results showed that FFA activated BMDMs co-culture impaired adipocyte insulin sensitivity, in which the phosphorylation levels of IRβ(Tyr1150/1151), IRS1(Ser636/639) and Akt (Ser473) under the stimulation of insulin were significantly lower than those cultured alone. In contrast, ponatinib weakened the activation of BMDM by FFA, thereby preserving the response of adipocytes to insulin signals under co-culture, which is reflected in the restored phosphorylation level of IRβ, IRS1 and Akt (Figures 8B–E).

## Discussion

Although the association between obesity and impaired insulin sensitivity has long been recognized, there are still subgroups of obese individuals whose insulin sensitivity is preserved (Appleton et al., 2013). These insulin-sensitive and insulin-resistant obese individuals have no significant difference in BMI, but WAT of the latter one has undergone severe adipose tissue remodeling. Adipose tissue remodeling refers to the dramatic changes in the cellular composition and microenvironment of adipose tissue triggered by excess calories, including hypoxia, fibrosis, and accumulation of pro-inflammatory macrophages (Vishvanath and Gupta, 2019). Similarly, A series of animal studies also showed that the expansion of “healthy” adipose tissue does not impair systemic insulin sensitivity. Slc2a4, Adipoq, mitoNEET, and Tnmd transgenic mice had an equal or even higher degree of obesity than their littermates, but their metabolic profile is healthier (Shepherd et al., 1993; Kim et al., 2007; Kusminski et al., 2012; Senol-Cosar et al., 2016). In our study, even if it failed to reduce the weight of obese mice or the mass of visceral adipose, ponatinib significantly attenuated the infiltration of macrophages and reduced pro-inflammatory cytokines in obese adipose tissue, and improved hepatic steatosis, hyperlipidemia and insulin resistance.

Leptin-deficient ob/ob mice are widely used in metabolic studies as a classic obese model. However, leptin seems to play a dual role in non-alcoholic fatty liver disease (NAFLD). It can prevent liver steatosis in the initial stage of NAFLD, but when the NAFLD progresses, it acts as a pro-inflammatory factor to promote non-alcoholic hepatitis (NASH) (Polyzos et al., 2015). In addition, although ob/ob mice can spontaneously form NAFLD under normal diet, the data showed that it is failed to further develop into typical hepatitis or hepatic fibrosis (Trak-Smayra et al., 2011). Unfortunately, this study could not further explore the effect of ponatinib on NASH due to the limitations of the ob/ob mouse model. For further study, high-fat diet (HFD) or methionine choline deficiency (MCD) models could be appropriate options (Soret et al., 2020).

Ponatinib is an orally active tyrosine kinase inhibitor that was originally designed to conquer the resistance mutations of BCR-ABL leukemia (O'Hare et al., 2009), but subsequent studies have shown that it has at least 30 high affinity targets (IC50 < 10 nm) (O'Hare et al., 2009). Due to these additional targets, recent growing evidence have indicated that ponatinib could exert benefits in the intervention of non-cancer diseases such as pulmonary hypertension, pulmonary fibrosis and cerebral cavernous malformations (Qu et al., 2015; Kang et al., 2016; Choi et al., 2018).

Here, ponatinib shows an inhibitory function in M1 macrophage polarization and adipose inflammation, however, the individual effects of each target of ponatinib are intriguing. In the high-affinity target spectrum of ponatinib, Csf1r undoubtedly plays a crucial role in the survival, proliferation and differentiation of myeloid cells, in addition, Fgr, Hck, Abl, and Lyn are also highly expressed in macrophages (Figure 6J) (Lowell, 2004; Tan et al., 2019). A recent study has shown that Fgr expression is up-regulated in macrophages of obese adipose tissue, and bone marrow Fgr deletion can inhibit adipose inflammation and metabolic dysfunction caused by M1 polarization of adipocyte macrophages in diet induced obesity (Acín-Pérez et al., 2020). In addition, although Hck and Abl is crucial for the activation and migration of macrophages (English et al., 1993; Cougoule et al., 2010; Zhou et al., 2020), the function of Hck and Abl in ATMs has not been investigated. In contrast to Fgr, Hck and Abl, Lyn is a unique member of the Src kinase family that negatively regulates innate immune response (Harder et al., 2001). Lyn agonist MLR-1023 has been reported to improve glucose tolerance in type 2 diabetic mice (Ochman et al., 2012). Taking together, the overall effect of ponatinib showed an improvement in the metabolic function of obese mice, but there are still some counterproductive targets in the target list of ponatinib. With the application of machine learning in drug design and optimization (Ekins et al., 2019; Kaiser et al., 2020; Walters and Barzilay, 2021), identifying a series of new compounds that were simultaneously optimized for on-target potency (e.g., Fgr, Hck, Abl) and off-target safety (e.g., Lyn) will provide more promising solutions for the pharmacological intervention of obesity-related metabolic disorders.

**FIGURE 9:**
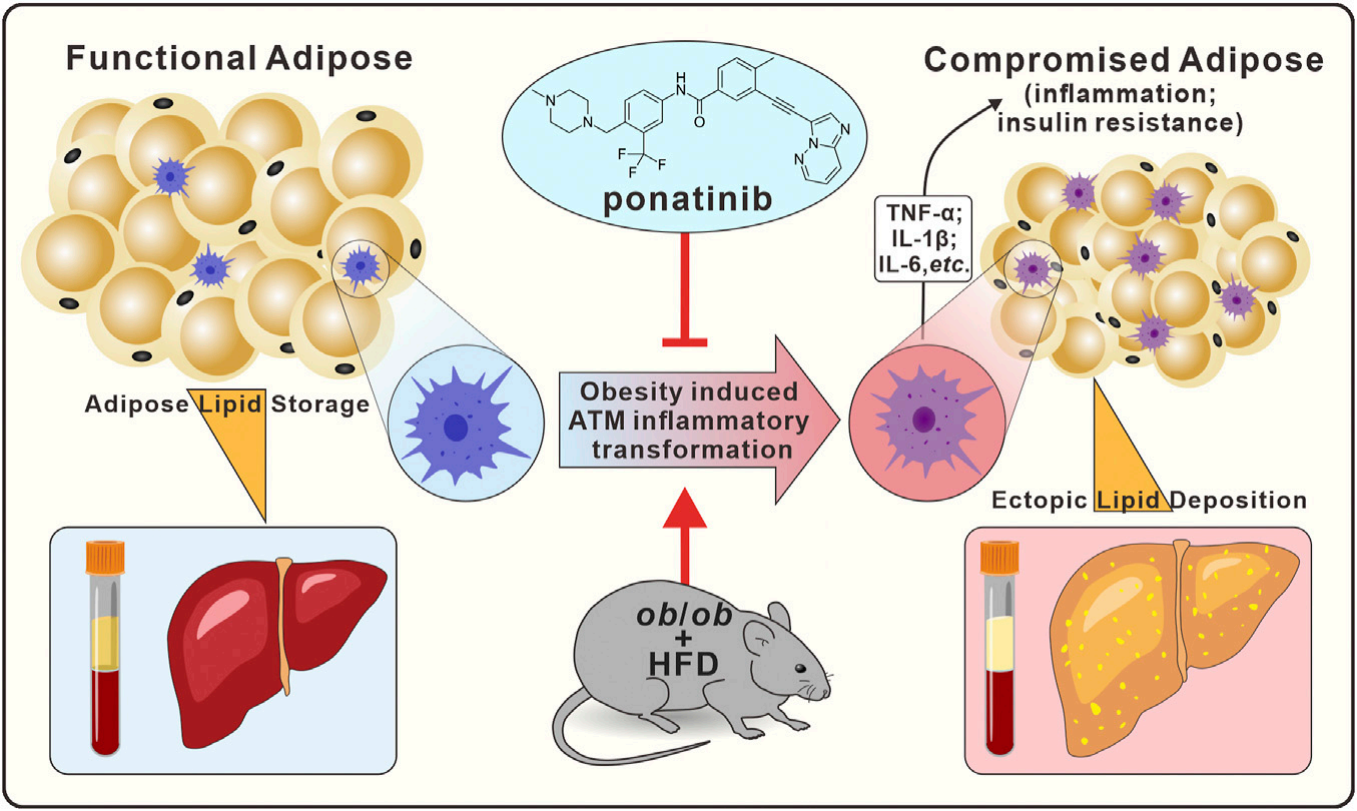
Graphical abstract. In ob/ob mice model, ponatinib inhibits obesity-induced adipose tissue macrophage inflammatory transformation, thereby inhibiting obesity adipose tissue inflammation and insulin resistance, accompanied by amelioration of ectopic lipid deposition in peripheral blood and liver.

In conclusion, our present results indicated that ponatinib had a therapeutic effect on metabolic dysfunction such as insulin resistance, dyslipidemia, NAFLD and adipose tissue inflammation. Ponatinib exerted these effects through inhibiting the inflammatory phenotypic transformation of ATM in the context of obesity. These data provided unequivocally evidence to support the therapeutic effect of ponatinib in obesity-related metabolic disorders, and provide a theoretical basis for the future application of multi-target tyrosine kinase inhibitors in the metabolic disorders (Figure 9, Graphical abstract).

## Data Availability

The raw data supporting the conclusions of this article will be made available by the authors, without undue reservation.
