# Supplementary material for: Ponatinib modulates the metabolic profile of obese mice by inhibiting adipose tissue macrophage inflammation
Source: Front Pharmacol. 2022 Nov 15;13:1040999. doi: 10.3389/fphar.2022.1040999 (PMC9705588; doi:10.3389/fphar.2022.1040999)
Supplement: Supplementary file 1 [file DataSheet3.PDF]

Figure 6E

Gel1 at different exposure times

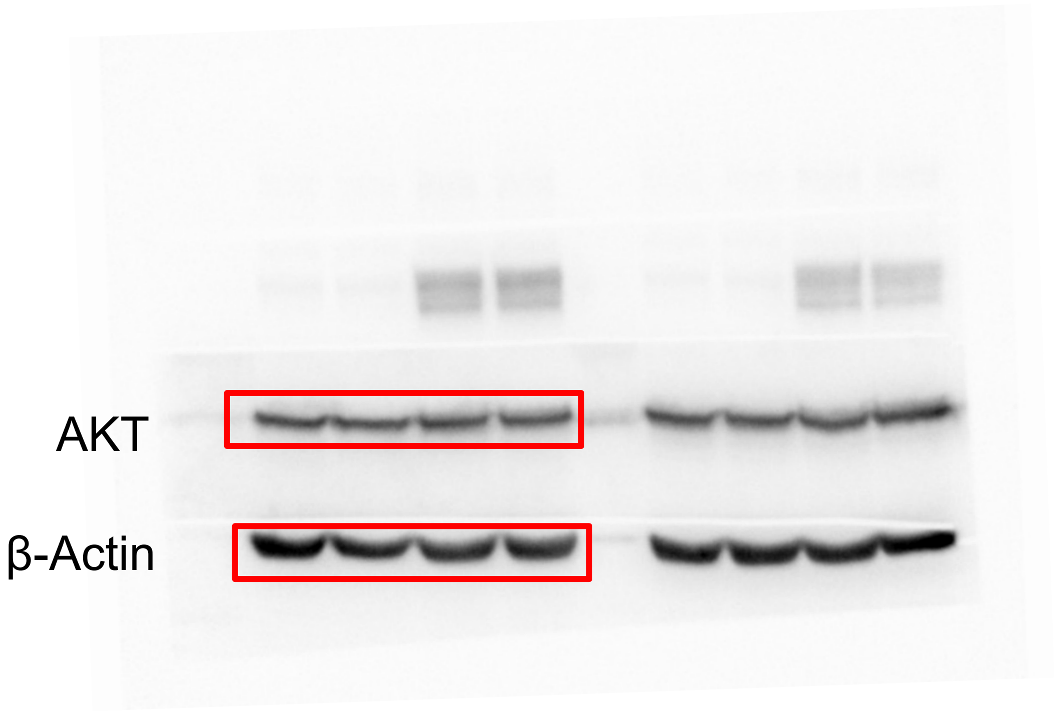

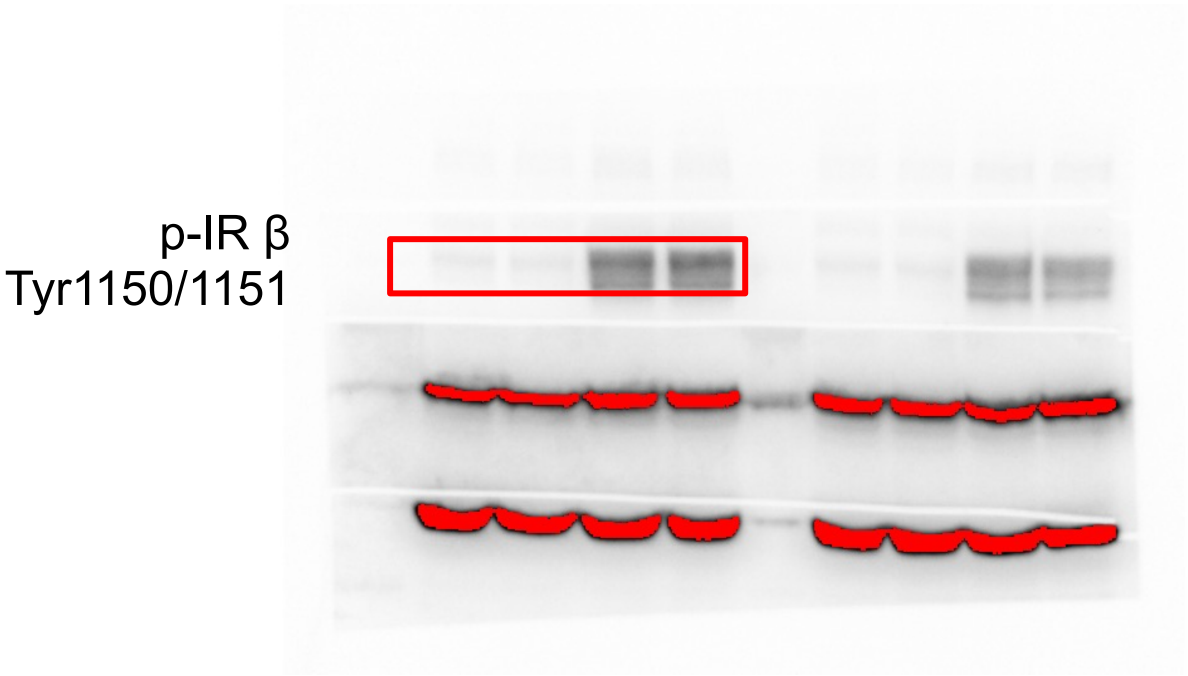

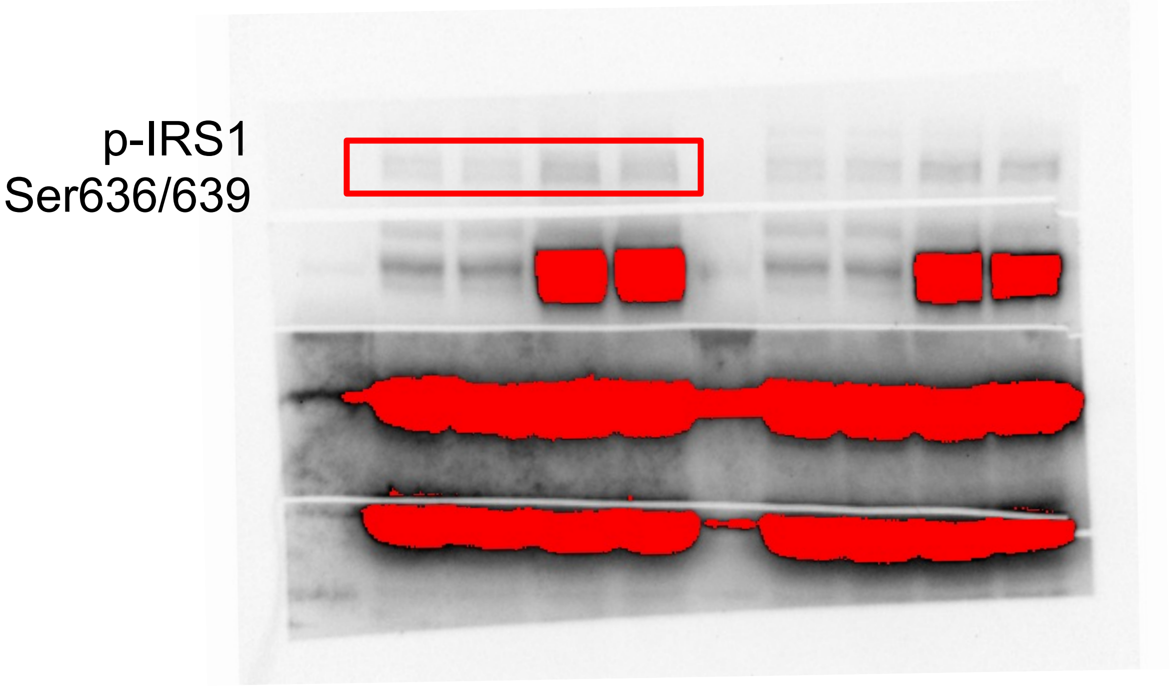

Figure 6E

Gel2 at different exposure times

IRS1

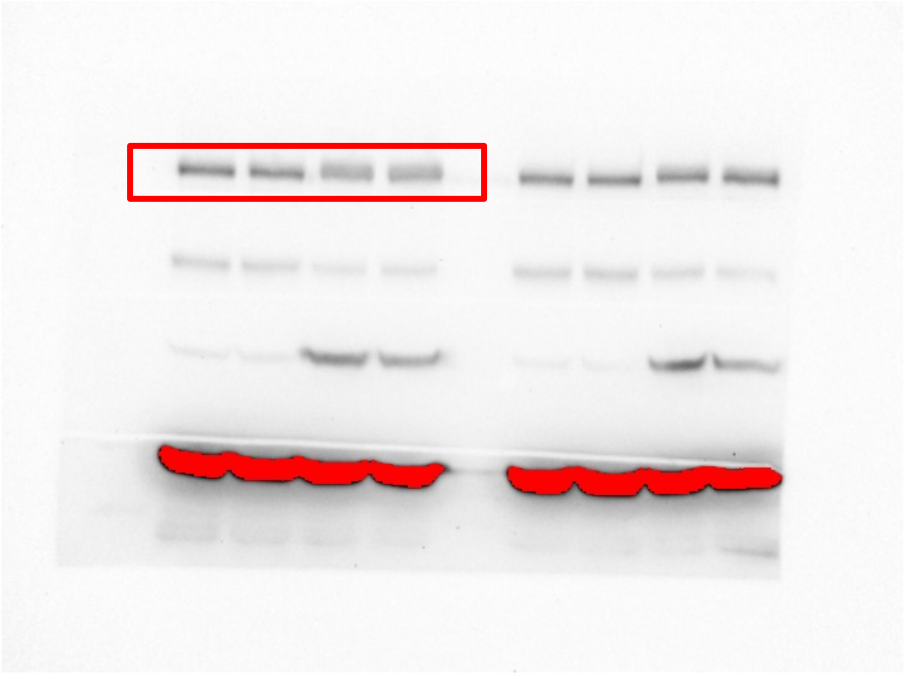

IR β

p-AKT
Ser473

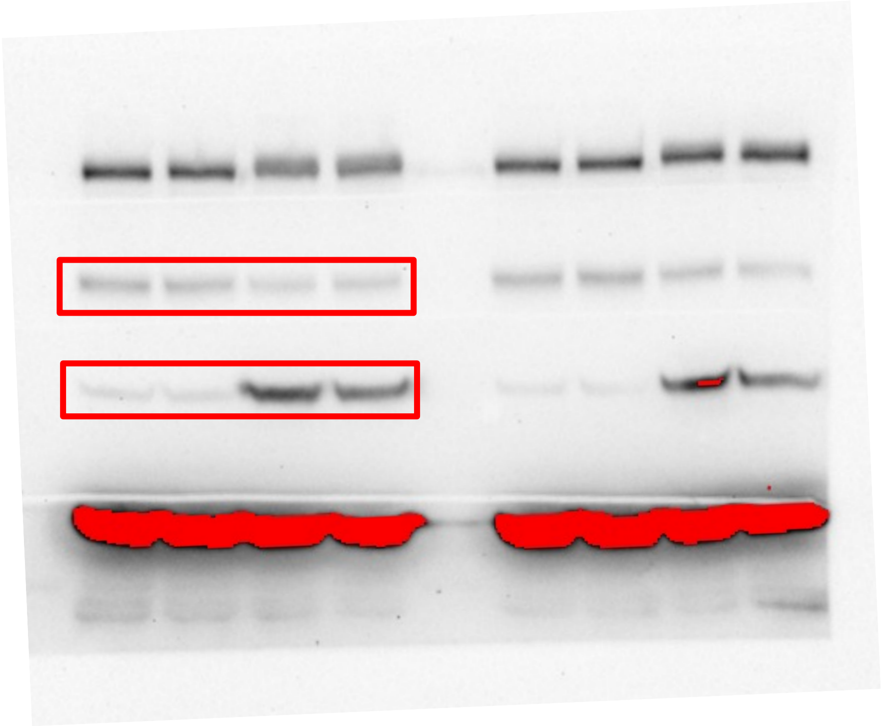

Figure 8B

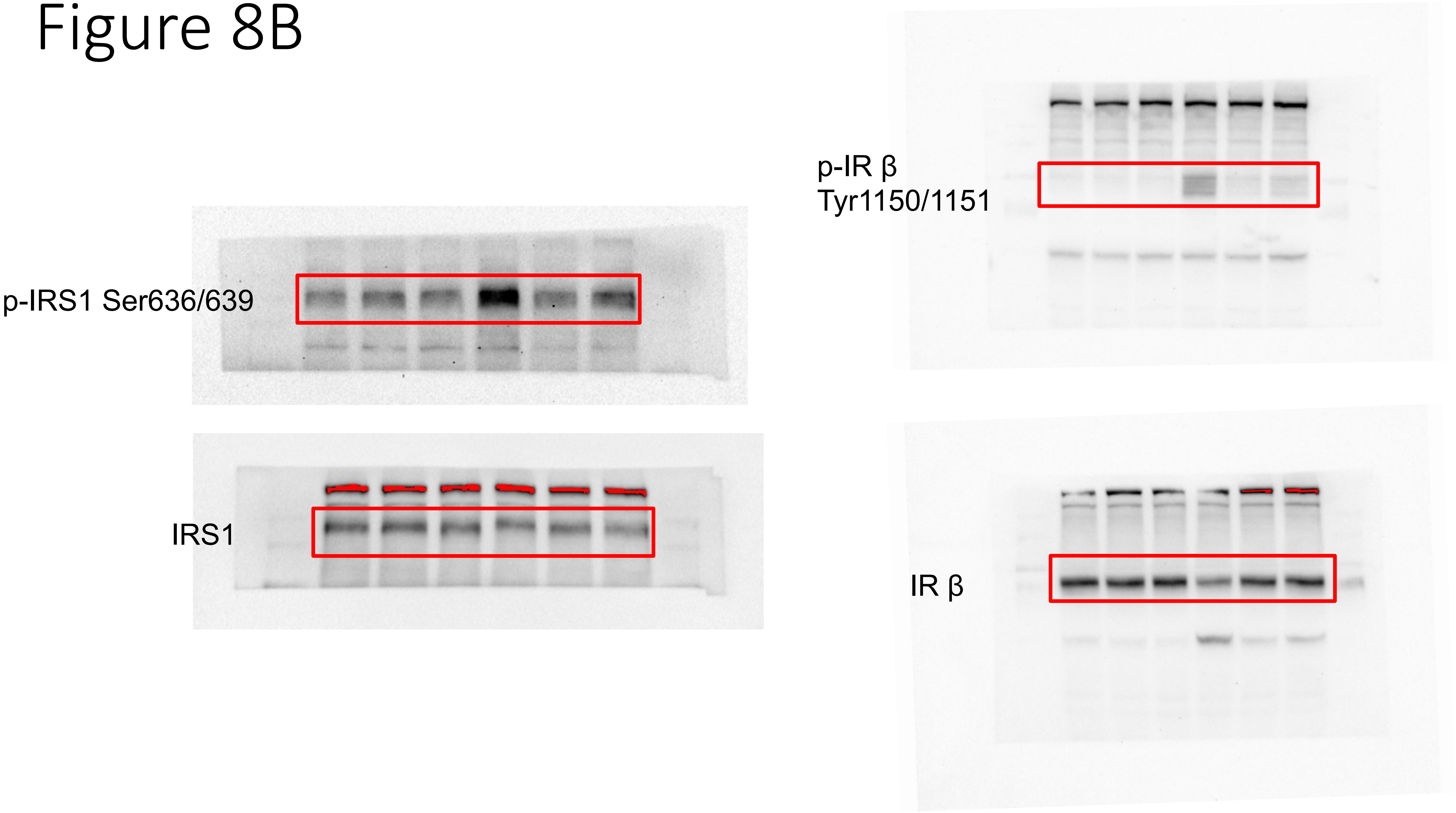

Figure 8B

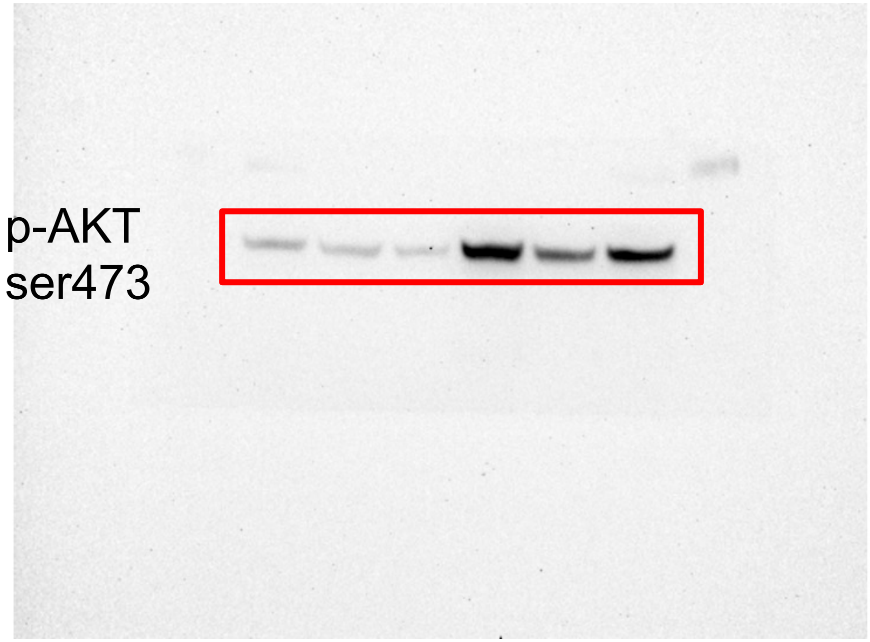

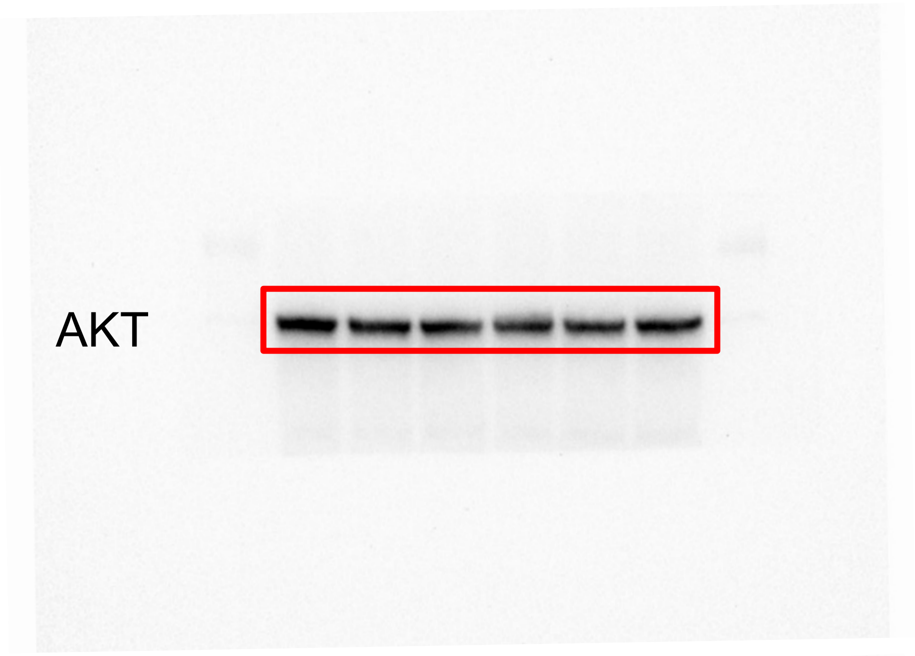

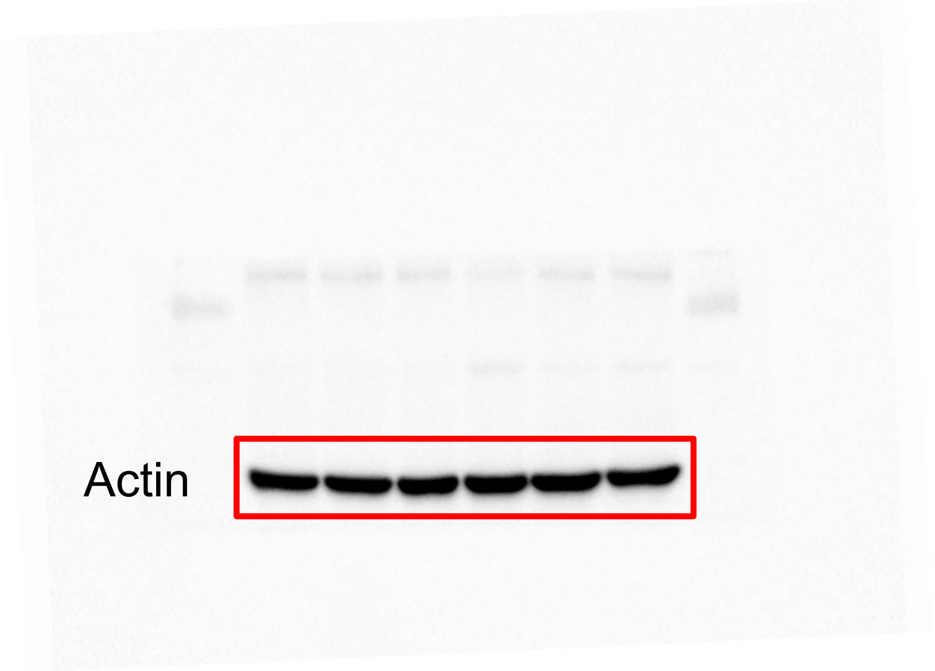

Figure 6J

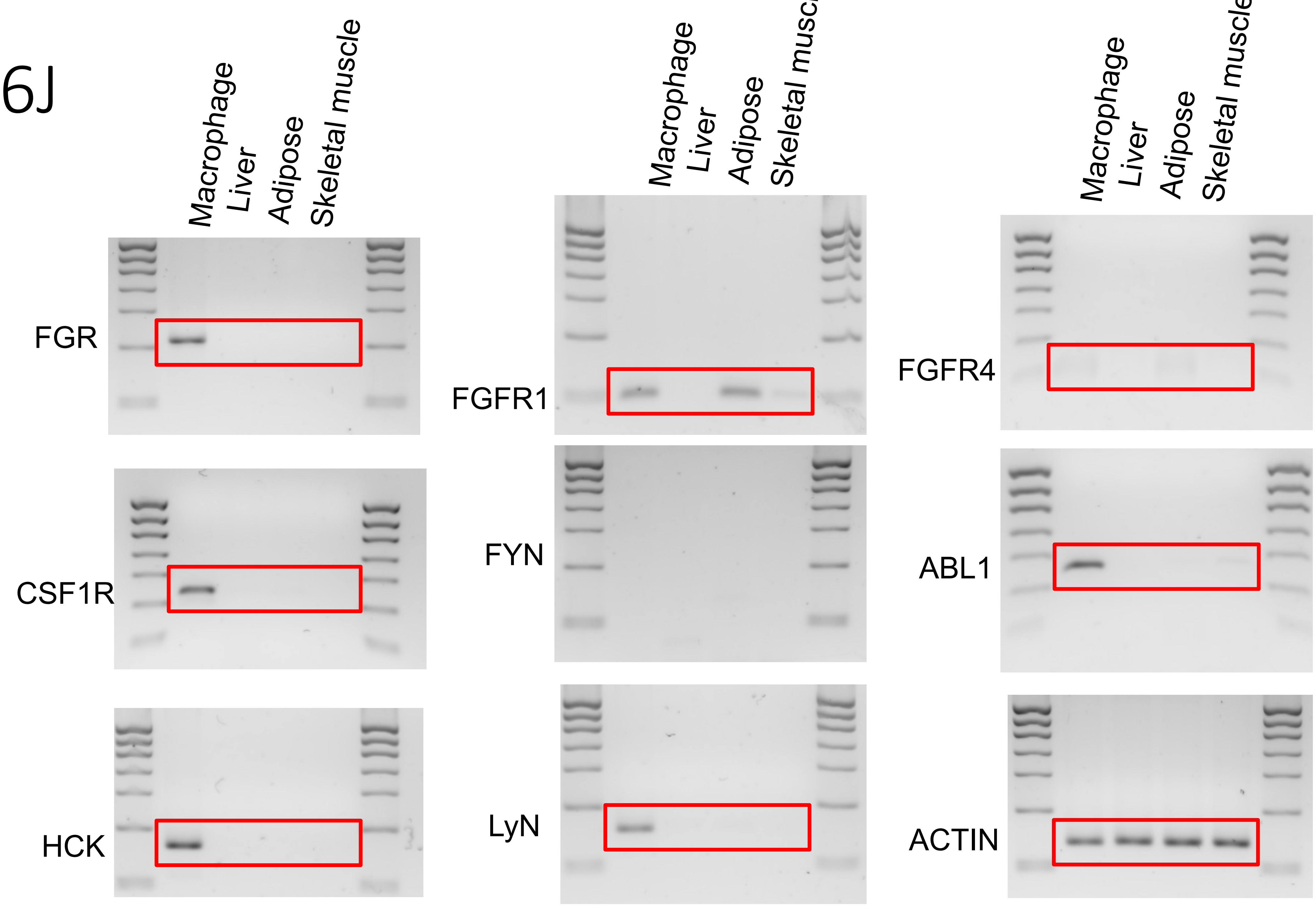

Macrophage
Liver
Adipose
Skeletal muscle
FGR
CSF1R
HCK
FGFR1
FYN
LyN
FGFR4
ABL1
ACTIN
